# Gestational oral low-dose estradiol-17β induces altered DNA methylation of *CDKN2D* and *PSAT1* in embryos and adult offspring

**DOI:** 10.1038/s41598-018-25831-9

**Published:** 2018-05-10

**Authors:** Vera A. van der Weijden, Veronika L. Flöter, Susanne E. Ulbrich

**Affiliations:** 1 ETH Zurich, Animal Physiology, Institute of Agricultural Sciences, Zurich, Switzerland; 2 Physiology Weihenstephan, Technical University of Munich, Munich, Germany; 3 Present Address: Department of Animal Physiology & Immunology, School of Life Sciences, Life Science Center Weihenstephan, Technical University Munich, Munich, Germany

## Abstract

Endocrine disrupting chemicals (EDC) interfere with the natural hormone balance and may induce epigenetic changes through exposure during sensitive periods of development. In this study, the effects of short-term estradiol-17β (E2) exposure on various tissues of pregnant sows (F_0_) and on day 10 blastocysts (F_1_) were assessed. Intergenerational effects were investigated in the liver of 1-year old female offspring (F_1_). During gestation, sows were orally exposed to two low doses and a high dose of E2 (0.05, 10, and 1000 µg/kg body weight/day). In F_0_, perturbed tissue specific mRNA expression of cell cycle regulation and tumour suppressor genes was found at low and high dose exposure, being most pronounced in the endometrium and corpus luteum. The liver showed the most significant DNA hypomethylation in three target genes; *CDKN2D*, *PSAT1*, and *RASSF1*. For *CDKN2D* and *PSAT1*, differential methylation in blastocysts was similar as observed in the F_0_ liver. Whereas blastocysts showed hypomethylation, the liver of 1-year old offspring showed subtle, but significant hypermethylation. We show that the level of effect of estrogenic EDC, with the periconceptual period as a sensitive time window, is at much lower concentration than currently presumed and propose epigenetics as a sensitive novel risk assessment parameter.

## Introduction

The environment can critically influence the complex regulation of the human body. Several environmentally present exogenous compounds are able to interfere with the endocrine system, possibly leading to adverse consequences to human health^1,2^. These substances are classified as ‘endocrine disrupting chemicals’ (EDC)^3^. Potent low dose effects of EDC, specifically estrogenic EDC as well as exogenous natural hormones, have been identified^4,5^. Exposure predominantly occurs through ingestion, inhalation, skin contact, and via the placenta of a pregnant female^6^. The molecular mechanisms of action involve the mimicking of regulatory pathways of endogenous hormones, such as oestrogens. Surface water has been found to be polluted with natural oestrogens such as estrone (E1), estradiol-17β (E2) and estriol (E3)^7^. These natural oestrogens, specifically E2, are globally released at varying, but polluting levels from wastewater treatment plants and effluents from livestock feedlots^7^. E2 is involved in various physiological functions such as growth, development and cell proliferation^8–10^. To explain observed cell proliferation and tumour initiation upon exposure to estrogenic substances, gene expression and epigenetic changes of cell cycle regulators, tumour suppressors, and methylation specific enzymes have been widely studied^11–17^.

Epigenetic changes have emerged as possible underlying mechanism through which EDC may exert their effect^18^. Epigenetics is defined as heritable changes in gene expression that are not due to changes in the DNA sequence^19^. One of the most widely explored and stable epigenetic mark is DNA methylation, which comprises the addition of a methyl group to the cytosine base of CpN dinucleotides^20,21^. Epigenetic changes at critical periods of development may permanently alter the epigenome in the germline, that can also be transmitted through generations^18^. When a pregnant female F_0_ is exposed, the subsequent two generations, namely the F_1_ and F_2_, are also directly exposed *in utero* or through the germline, respectively, and may exhibit intergenerational epigenetic inheritance. Any observed effects in subsequent generations are known as transgenerational epigenetic inheritance without any direct exposure^22^.

A far greater concern than direct effects of EDC - with large implications for human health - is that alterations to the epigenome may not cause immediate phenotypic effects, but remain as molecular fingerprints; only leading to a phenotype long after exposure or in subsequent generations^23,24^. Nonetheless, the current understanding of the link between E2 exposure during critical periods of early development and intergenerational epigenetic changes is limited.

In the present study, we aimed at

1. Studying the effects of a short-term low dose E2 exposure on tissue specific mRNA expression and DNA methylation in pregnant sows,
2. Assessing the methylation changes in gestationally exposed blastocysts to identify whether the exposure during early embryo development already induces a fingerprint to these low E2 doses,
3. Investigating whether changes in DNA methylation as observed in the embryos were also present in the one-year old adult offspring that had been exposed during the entire pregnancy.

**Figure 1:**
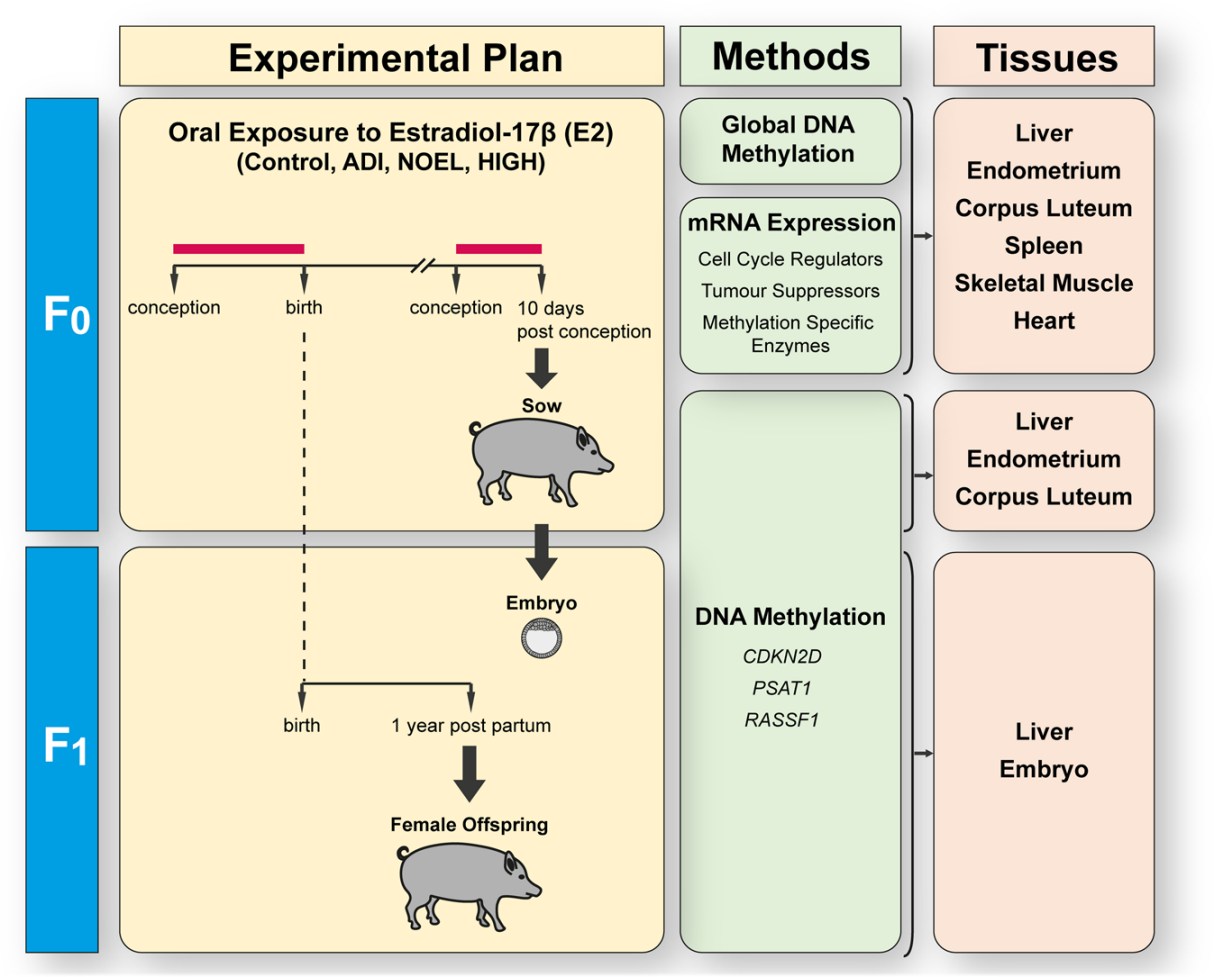
Summary of the workflow undertaken in this study. The experimental plan indicates the oral exposure to estradiol-17β (E2) (indicated with red bars) at concentrations of 0, 0.05, 10 and 1000 μg/kg body weight/day for the control, ADI – ‘acceptable daily intake level’, the NOEL- ‘no observed effect level’, and a HIGH dose, respectively. Pregnant sows (F_0_) were exposed to treatments twice a day from conception to birth and again from conception to day 10 post-conception, after which they were slaughtered one hour after the last treatment. Embryos (F_1_) were flushed from the uterus directly after slaughter. Female offspring (F_1_) were sampled at one year of age during the diestrous phase of the oestrous cycle.

To that end, two low doses, namely the acceptable daily intake (ADI) and a dose close to the no observed effect level (NOEL) (0.05 and 10 μg/kg body weight/day, respectively) and a high dose (HIGH) (1000 μg/kg body weight/day) of E2 were orally applied to sows as described previously^25^ (Fig. 1 and Supplementary Fig. S1 and Supplementary Text). Targeted gene expression analysis of relevant genes was undertaken, followed by the analysis of global DNA methylation changes in different tissues, such as liver, endometrium, corpus luteum, heart, spleen and skeletal muscles of exposed pregnant females (F_0_). Furthermore, gene specific DNA methylation changes were analysed in target regions of selected genes, namely Cyclin Dependent Kinase Inhibitor 2D (*CDKN2D*), Phosphoserine Aminotransferase 1 (*PSAT1*) and Ras Association Domain Family Member 1 (*RASSF1*). The gene specific DNA methylation was analysed in the liver, endometrium and corpus luteum of F_0_ sows, day 10 embryos (blastocysts) and the liver of one-year old female offspring (both F_1_) (Fig. 1). *CDKN2D*, a member of the INK4 family, encodes for p19INK4d and is involved in the regulation of cell growth^26^. The promoter region of *CDKN2D* has previously been shown to be differentially methylated, influencing its transcriptional levels^26^. *PSAT1* is an enzyme that catalyses the serine biosynthesis pathway, with documented evidence of its cell proliferation activity, including cell cycle progression and tumorigenesis^27,28^. The tumour suppressor gene *RASSF1* is known to induce cell cycle arrest and senescence in the G1 phase^29^. In addition, *RASSF1* has been shown to be epigenetically silenced in various tumours and upon E2 exposure^16,30^.

Our data shows that E2 doses as low as the acceptable daily intake cause changes in both the gene expression and DNA methylation profile of selected targets, underlining that epigenetics could serve as a sensitive novel parameter for risk assessment of estrogenic EDC.

## Results

### E2 induces tissue specific gene expression changes of cell cycle regulators and tumour suppressor genes in F_0_ tissues

**Figure 2:**
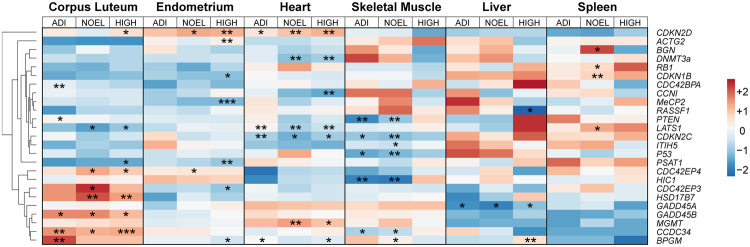
Heat map using hierarchical clustering of differentially expressed genes (DEG) in F_0_ tissues. Left to Right: Corpus luteum, endometrium, heart, skeletal muscle, liver, and spleen, where rows represent genes and columns represent the centred mean log-fold changes per treatment group compared to the control (0.05, 10, and 1000 μg/kg body weight/day represented by ADI, NOEL, and HIGH dose, respectively). *, **, and *** indicate statistical significance with respect to the control group at p < 0.05, p < 0.01, and p < 0.001, respectively (n = 4–6 per group).

The gene expression analyses were carried out in F_0_ liver, endometrium, corpus luteum, heart, spleen, and skeletal muscle using 57 selected estrogen-related target genes (Supplementary Table S1). Overall, 25 genes were found to be differentially expressed in various tissues and treatment groups as compared to the control group (Fig. 2). These included 11 cell cycle regulators (*CDKN2D, CDC42EP4, GADD45A, MGMT, GADD45B, CCDC34, CDC42EP3, CDKN1B, CCN1, CDC42BPA*, and *CDKN2C)*, 10 tumour suppressor genes (*HIC1, BGN, RB1, RASSF1, LATS1, PTEN, P53, ITIH5, PSAT1*, and *ACTG2*), two methylation specific genes (*DNMT3a* and *MeCP2*), one gene associated with aberrant expression in cancer (*BPGM*), and one gene functionally associated with the biosynthesis of sex steroids (*HSD17B7*). Most differentially expressed genes (DEG) were observed in the corpus luteum followed by the endometrium, heart and skeletal muscle.

Amongst the DEG, the cell cycle regulator *CDKN2D* was most highly upregulated in the NOEL and HIGH dose group in the endometrium (9.0-fold and 15.2-fold, respectively). *CDKN2D* also showed a significant upregulation in the HIGH dose group of corpus luteum (9.7-fold), and a dose-dependent increase of its expression in heart with 4.1-fold, 6.2-fold and 10.6-fold for ADI, NOEL, and HIGH, respectively. The most downregulated DEG (12.5-fold) was the tumour suppressor gene hypermethylated in cancer 1 (*HIC1)*, in skeletal muscle in the NOEL dose group. The expression of *PSAT1* was significantly downregulated in the HIGH dose in the corpus luteum and endometrium (4.3-fold and 2.4-fold, respectively). *RASSF1* was not affected in either groups nor tissues, with exception of the HIGH dose group of the liver (1.3-fold downregulated) (Supplementary Table S2).

### E2 does not significantly influence global DNA methylation in F_0_ tissues

The analysis of the global DNA methylation in the F_0_ tissues showed a tendency of hypomethylation in the HIGH dose group of spleen (2%), skeletal muscle (2%), heart (1%), and in all dose groups of corpus luteum (1% in ADI, NOEL and HIGH) as compared to controls. None of these differences reached statistical significance (Supplementary Table S4).

### E2 induces DNA hypomethylation of *CDKN2D, PSAT1*, and *RASSF1* in F_0_ liver

Gene specific DNA methylation analysis was carried out on selected CpN sites in the putative promoter region of *CDKN2D*, the coding region of the first exon of *PSAT1*, and the 5′ untranslated region (UTR) of the first exon of *RASSF1* (Supplementary Table S5).

**Figure 3:**
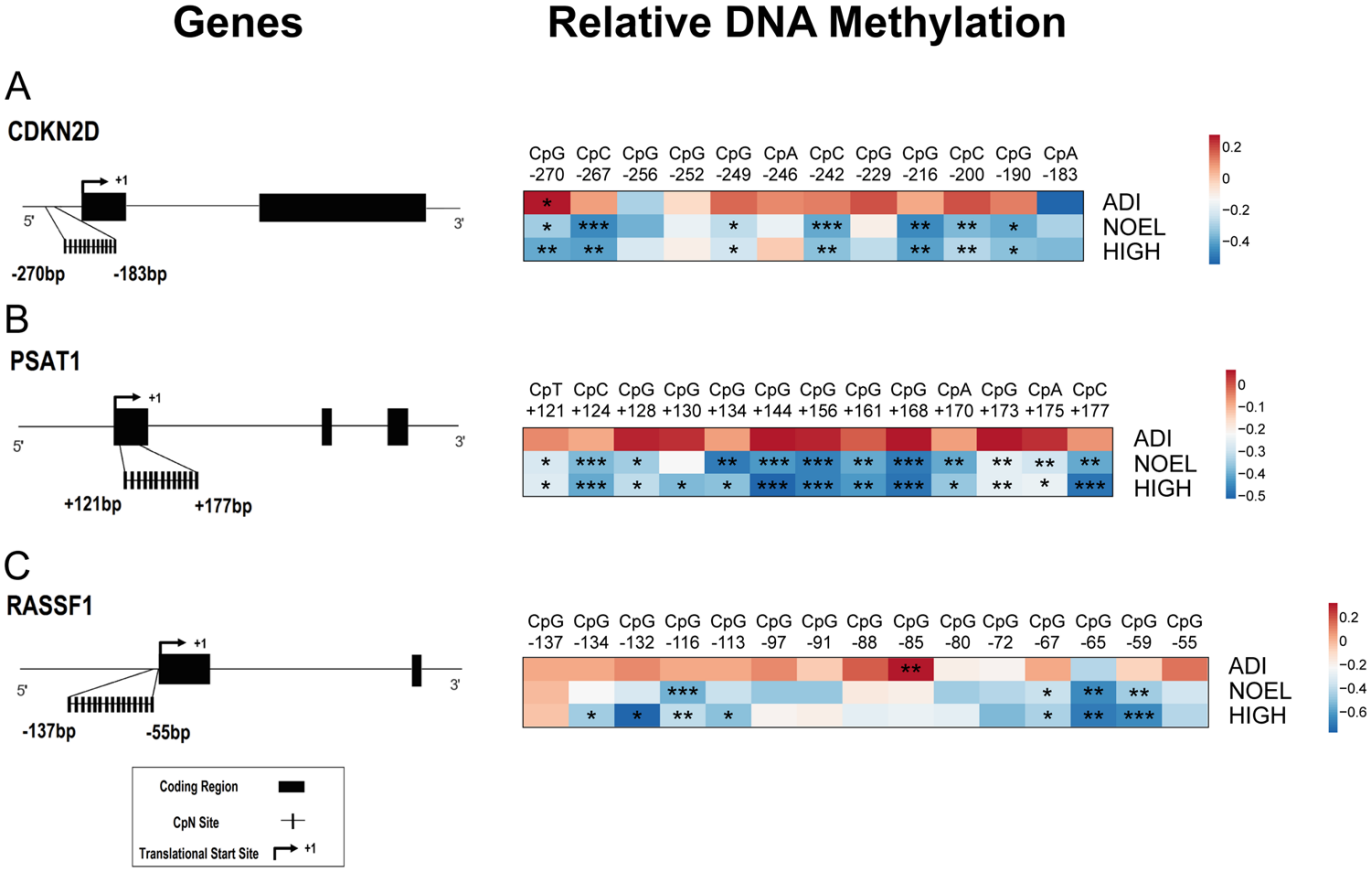
Heat maps showing gene-specific DNA hypomethylation at CpN sites of NOEL and HIGH dose groups of F_0_ liver. Each column of the heat maps shown represents a CpN site analysed with its position with respect to the translational start site (indicated by +1), while each row represents the log_2_ methylation relative to control (0.05, 10, and 1000 μg/kg body weight/day represented by ADI, NOEL, and HIGH, respectively). *, **, and *** indicate statistical significance compared to the control group at p < 0.05, p < 0.01, and p < 0.001 respectively (n = 4–6 per group). (**A**) Gene diagram of *CDKN2D*, indicating the CpN sites analysed in the putative promoter region. (**B**) Gene diagram of *PSAT1* indicating the CpN sites analysed in the coding region of the first exon. (**C**) Gene diagram of *RASSF1* indicating the CpN sites analysed in the first exon.

The E2 exposure induced subtle, but significant hypomethylation in several CpN sites analysed for all three genes in the NOEL and HIGH dose groups of F_0_ liver (Fig. 3). The most pronounced and statistically significant hypomethylation compared to control in each of the three genes analysed was in the HIGH group with a 1.7-fold lower methylation for *RASSF1* (CpG −132), followed by a 1.4-fold lower methylation in the NOEL group for *RASSF1* (GpC −267) and the HIGH group for *PSAT1* (CpG +144).

### E2-mediated hypomethylation of the *CDKN2D* promoter region in F_0_ tissues and F_1_ embryos, but hypermethylation in adult F_1_ liver

**Figure 4:**
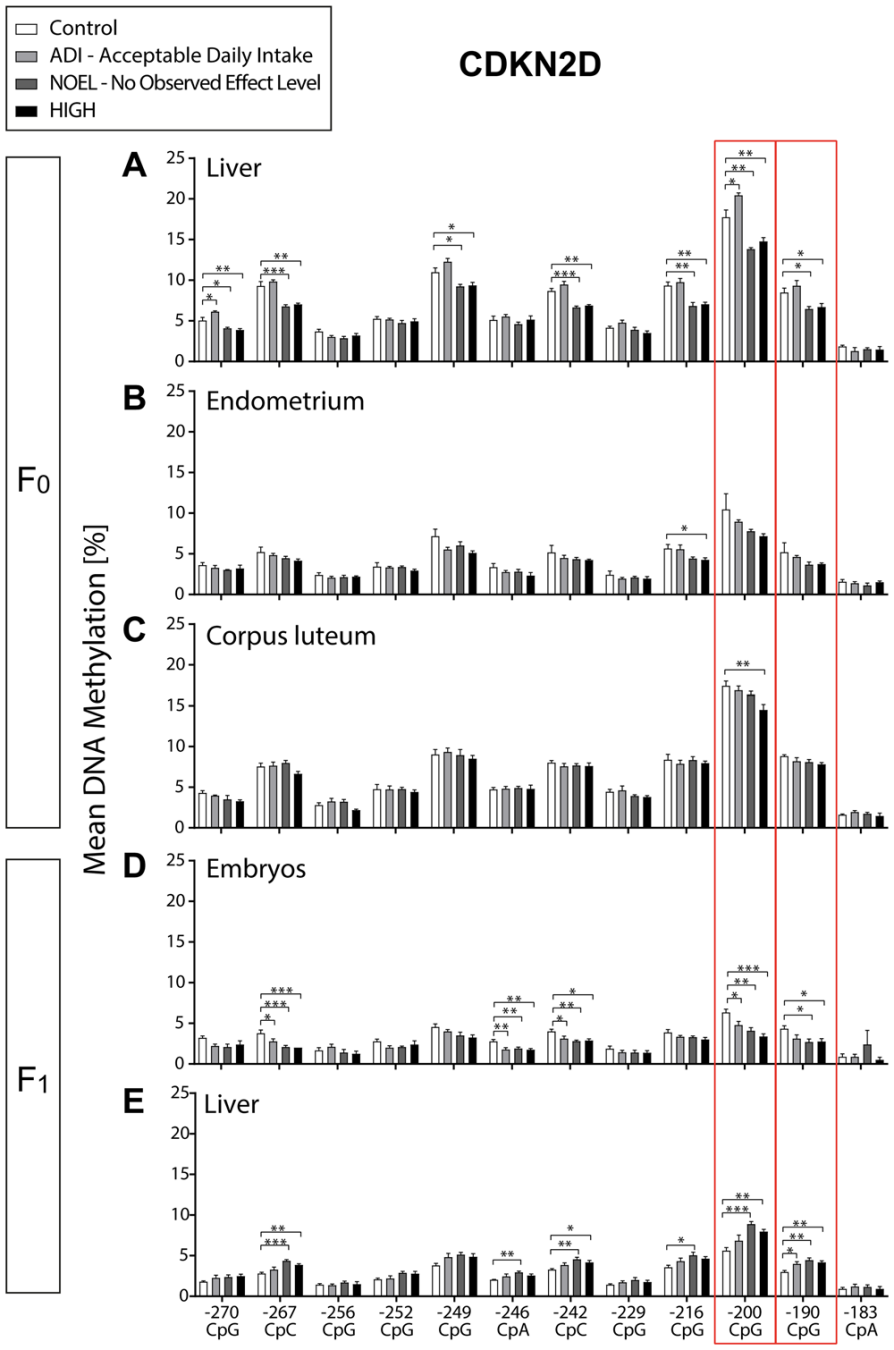
E2 induced subtle, but consistent DNA hypomethylation in the *CDKN2D* putative promoter region in F_0_ liver, endometrium, and corpus luteum, and F_1_ embryos, but hypermethylation in liver of F_1_ adult gilts. Bar charts represent mean ± SEM of % DNA methylation per group in different shades of grey (0.05, 10, and 1000 μg/kg body weight/day represented by ADI, NOEL, and HIGH dose, respectively). *, **, and *** indicate statistical significance with respect to the control group at p < 0.05, p < 0.01, and p < 0.001, respectively (n = 4–6 per group for tissue samples, and n = 8–10 for embryos). (**A**) F_0_ liver DNA hypomethylation at CpN −270, −267, −249, −242, −216, −200, and −190. (**B**) F_0_ endometrium DNA hypomethylation at CpG −216. (**C**) F_0_ corpus luteum DNA hypomethylation at CpG −200. (**D**) F_1_ embryo DNA hypomethylation at CpN −267, −246, −242, −200, and −190 (**E**) F_1_ liver DNA hypermethylation at CpN −267, −246, −242, −216, −200, and −190.

The differentially methylated CpN sites in the promoter region of *CDKN2D* primarily showed hypomethylation in exposed versus control animals. This effect was more pronounced in the liver than in any of the reproductive tissues under analysis (Fig. 4). Eventhough the most pronounced DNA methylation differences were observed in the F_0_ liver, there was no significant correlation to the subtle downregulation in mRNA expression (Supplementary Table S6). Similar to the F_0_ tissues, the blastocysts showed only hypomethylated CpN sites. Interestingly, an opposite pattern of hypermethylation was revealed in the liver of F_1_ adult animals. Focusing on CpG −200, a trend towards a dose-dependent decrease was observed for the reproductive tissues, with statistically significant hypomethylation in the corpus luteum of the F_0_ HIGH dose group. Even though the methylation differences were very subtle, the DNA methylation at this site significantly correlated with the transcription in the corpus luteum (R = 0.56 p = 0.01) (Supplementary Table S6). In addition, the DNA methylation of CpC −242 and CpG −190 significantly correlated with the transcription in the corpus luteum (R = 0.49, p = 0.029 and R = 0.60 p = 0.005, respectively) (Supplementary Table S6). The DNA methylation in the endometrium was only lower in the HIGH dose group at GpG −216 (Fig. 2), whereas mRNA expression was increased in the NOEL and HIGH dose group. The F_1_ embryos showed statistically significant hypomethylation at CpG −267, CpA −246, CpC −242, and CpG −200 for all doses, and at CpG −190 in the NOEL and HIGH dose. Opposing the methylation pattern in the maternal (F_0_) liver and the F_1_ embryos, the adult F_1_ liver showed statistically significant hypermethylation at CpC −267, CpA −246, CpC −242, CpG −216, CpG −200, and CpG −190.

### E2-mediated hypomethylation of the coding region of PSAT1 in F_0_ liver and hypermethylation in F_1_ liver

**Figure 5:**
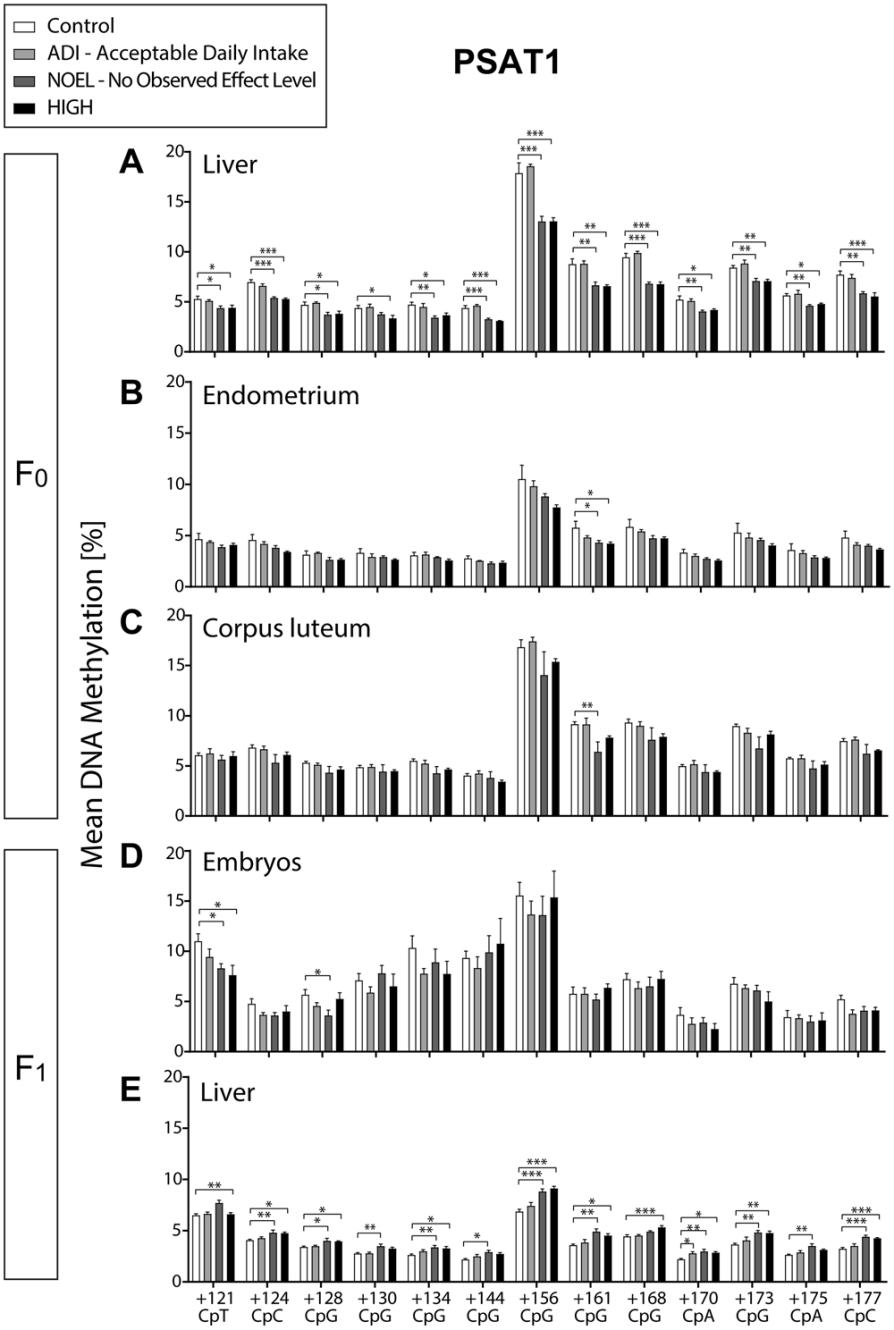
E2 induced consistent DNA hypomethylation in the *PSAT1* coding region (first exon) in F_0_ liver, endometrium, and corpus luteum, and F_1_ embryos but hypermethylation in corresponding CpG sites of the liver of adult F_1_ animals. Bar charts represent mean ± SEM of % DNA methylation per group in different shades of grey (0.05, 10, and 1000 μg/kg body weight/day represented by ADI, NOEL, and HIGH dose, respectively). *, **, and *** indicate statistical significance with respect to the control group at p < 0.05, p < 0.01, and p < 0.001, respectively (n = 4–6 per group for tissue samples, and n = 8–10 for embryos). (**A**) F_0_ liver DNA hypomethylation at all analysed CpN sites. (**B**) F_0_ endometrium DNA hypomethylation at CpN +161. (**C**) F_0_ corpus luteum DNA hypomethylation at CpG +161. (**D**) F_1_ embryo DNA hypomethylation at CpN +121, and +128 (**E**) F_1_ liver DNA hypermethylation at all analysed CpN sites.

All 13 CpN sites in the coding region of the PSAT1 first exon showed statistically significant hypomethylation in the F_0_ liver in NOEL and/or HIGH dose animals (Fig. 5). CpG +161 was significantly hypomethylated in the F_0_ endometrium and corpus luteum in the NOEL and HIGH dose animals, and in NOEL dose animals, respectively. Except for CpA +170 (R = −0.47 p = 0.044) in the liver, there was no correlation between methylation and gene expression at any of the sites analysed (Supplementary Table S6). In the F_1_ blastocysts CpT +121 and CpG +128 showed differential methylation patterns, which were comparable to the F_0_ liver. Moreover, while CpG +128 was hypomethylated in blastocysts, it was found to be hypermethylated in the 1-year post-partum F_1_ animals. In contrast to the hypomethylation of all 13 CpN sites in the F_0_ liver, in the F_1_ liver all 13 CpN sites were hypermethylated.

### E2 exposure alters DNA methylation of the 5′ UTR of *RASSF1* in F_0_ liver correlating with *RASSF1* gene expression

**Figure 6:**
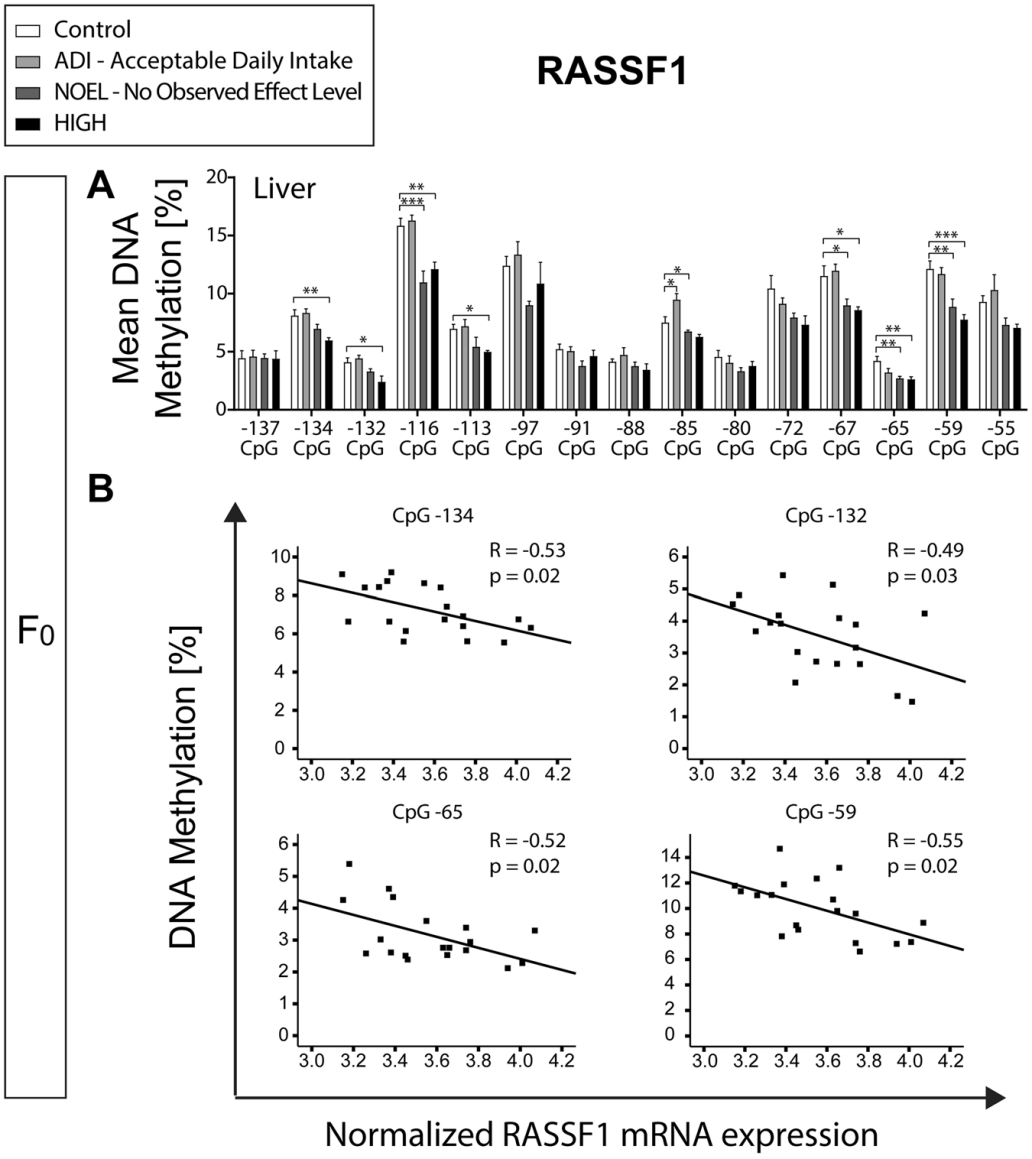
E2 induced DNA hypomethylation in the *RASSF1* 5′ UTR of the F_0_ liver. Bar charts represent mean ± SEM of % DNA methylation per group in different shades of grey (0.05, 10, and 1000 μg/kg body weight/day represented by ADI, NOEL, and HIGH dose, respectively). *, **, and *** indicate statistical significance with respect to the control group at p < 0.05, p < 0.01, and p < 0.001, respectively (n = 4–6 per group). (**A**) Liver DNA methylation showing most pronounced hypomethylation at CpG −134, −132, −116, −113, −85, −72, −67, −65, and −59. (**B**) Significant correlation of mRNA expression of RASSF1 with its methylation levels at CpG −134, −132, −65, and −59.

Eight of the 15 CpG sites analysed in the 5′ untranslated region of *RASSF1* first exon showed statistically significant hypomethylation in the F_0_ liver. The DNA methylation assessed at CpG −134, CpG −132, CpG −72, CpG −65, and CpG −59 significantly correlated with *RASSF1* liver gene expression (Fig. 6 and Supplementary Table S6), i.e. higher methylation was correlated with higher expression. Besides the F_0_ liver, neither endometrium nor corpus luteum of the F_0_ animals showed differential methylation, and there was no correlation between methylation and gene expression (Supplementary Fig. S4 and Table S6). In F_1_ blastocysts, the CpG −55 was significantly hypomethylated in ADI and NOEL exposed animals. The F_1_ liver did not show any differential DNA methylation (Supplementary Fig. S4).

## Discussion

Our results indicate that continuous oral exposure to a low dose E2 throughout pregnancy, currently considered as safe in the range of human “acceptable daily intake” (ADI) and the “no observed effect level” (NOEL)^31^, induces aberrant gene expression and DNA methylation. Given the high degree of physiological and anatomical similarity between pigs and humans, specifically the low circulating endogenous oestrogen concentrations during the periconceptional period which opposes the later pronounced synthesis of oestrogens by the placenta during pregnancy, the pig resembles human physiology^32^. Therefore, the pig was chosen as model organism to investigate the effects of gestational exposure to an additional low-dose estrogenic substance, potentially mimicking intergenerational epigenetic changes in women. The observed molecular changes were apparent in treated mothers, and in both her developing blastocysts and one-year old offspring. These findings are of particular note, as the circulating levels of E2 in animals under both low-dose treatments remained similar to controls^25^.

The tissue-specific differential gene expression of cell cycle regulators and tumour suppressors highlights the tissue-specific transcriptional E2 responsiveness^33^. The female reproductive tract is known to be a major target of estrogenic activity due to the high expression of oestrogen receptors^34,35^. Moreover, recent studies have shown that skeletal muscle is likewise an E2-responsive tissue^36^. *CDKN2D* showed the highest upregulation in reproductive tissues and heart, whereas *HIC1* was most pronouncedly downregulated in skeletal muscle. In addition, *PSAT1* was significantly downregulated in the corpus luteum. As possible explanation for the pronounced upregulation of *CDKN2D* as cell cycle inhibitor in heart and reproductive tissues we propose a negative feedback mechanism, i.e. repression of cell proliferation, of E2-induced cell hyperproliferation^37^. In addition, *HIC1*, the most pronouncedly downregulated DEG, is a known tumour suppressor gene^38^. Interestingly, the expression of *CDKN2D, HIC1*, and *PSAT1* have been linked to the transcriptional factor of cell cycle regulation *E2F1*^27,39,40^. The latter, in turn, has been shown to be mediated by E2^41^. Therefore, the results may indicate a complex interplay of molecular factors leading to the tissue specific differential expression of *CDKN2D*, *HIC1*, and *PSAT1* at high, as well as at low-dose E2 exposure. Lastly, *RASSF1* was lower expressed in the liver of the HIGH dose group. Lower expression of *RASSF1* has been reported to play a role in tumorigenesis^30^. In addition to that, silencing of *RASSF1* gene expression has been linked to *de novo* hypermethylation; *RASSF1* hypermethylation has previously been found in human tumours, including liver cancer^30^, indicating the HIGH dose of E2 as potential cancer-inducing risk factor.

The gene specific DNA methylation of selected regions in the *CDKN2D, PSAT1* and *RASSF1* genes revealed subtle, but particularly interesting changes. In terms of number of CpG sites displaying a significant difference in DNA methylation, the liver was the most affected tissue.

The methylation state of different regions within a gene is known to differentially impact gene expression. In this respect, promoter hypermethylation and gene silencing have been studied most extensively^42^. A negative correlation between mRNA transcription and DNA methylation of the promoter region of *CDKN2D* has recently been shown^26^. Likewise, the analysed sites CpG −200 and CpG −190 in the putative promoter region of *CDKN2D* in the corpus luteum of F_0_ sows showed subtle changes in the methylation status that negatively correlated with its gene expression. On the contrary, the methylation status of neither CpG −200 nor CpG −190 in the liver did not. Specific methylation patterns of known transcriptionally silenced tumour suppressor genes in primary tumours have been shown previously; the *CDKN2D* promotor was differentially methylated in different types of colorectal cancer, but not specifically correlated with gene expression^43^. Thus, while our results clearly indicate tissue-specific methylation, the methylation independent gene expression which probably underlies the lack of correlation between methylation and gene expression in the liver, remains to be further elucidated.

Besides the promotor methylation as regulator of gene expression, regions close to the transcriptional start site such as first exons, CpG islands and CpG shores have also been shown to influence gene expression^44^. In both the *PSAT1* first exon coding region and the *RASSF1* first exon 5′UTR, several CpN sites were significantly hypomethylated in the liver of F_0_ animals across treatment groups. Despite the consistent hypomethylation of *PSAT1*, there was no correlation with the gene expression of *PSAT1* in the liver of F_0_ sows. We assume that the location of the *PSAT1* target region, i.e. the first exon coding region, might explain the absence of any correlation between methylation and gene expression. The methylation levels of *RASSF1* in the NOEL and HIGH dose groups at CpG sites −134, −132, −65, and −59 significantly positively correlated with its gene expression. Our data underline the importance of CpG islands in the promotor region (*CDKN2D)* and the first exon 5′UTR (*PSAT1*) as gene expression regulators^45,46^.

In general, the changes in DNA methylation in our study were very subtle compared to differences found during development, between tissues and in normal versus cancer cells^47^, raising the question whether methylation status solely resembles a molecular fingerprint of exposure, or that it emerges a precursor for development of disease later in life and/or evokes transgenerational epigenetic effects^46^. Developmental-stage specific DNA methylation changes might hinder the assessment of local DNA methylation during early embryo development, but it has previously been shown that the embryoblast and trophectoderm have reached relatively stable levels of DNA methylation in day 10 pig embryos^48,49^. In addition, an increased number of studies have shown that exposure to environmental compounds during pregnancy can affect epigenetic marks in developing offspring, thereby priming individuals for the development of disease later in life^50^. Previously, we have shown that the low-dose E2 exposure during pregnancy increased the body fat percentage of prepubertal male offspring^25^, and slightly changed bone density parameters of female offspring^5^. In addition, we have reported a subtle but significant hypermethylation of the *BGN* gene in prostate of F_1_ male offspring upon *in uter*o exposure to HIGH E2^51^. In the current study, we provide evidence that the impact of the low-dose E2 treatment during pregnancy on DNA methylation changes were evident in F_0_ tissues as well in female offspring and, most importantly, occurred as early as the blastocyst stage. Whether the exposure to E2 directly imposed a molecular fingerprint on the embryo or indirectly by perturbing the uterine environment, which in turn affected the susceptible preimplantation conceptus, remains to be shown. One drawback of the current study is that the sows (F_0_) analysed for a short-term treatment effect had received E2 twice, once during a whole gestation period and again for 10 days, with a non-treated period of at least 8 months in between. Driven by the 3R (replacement, reduction, and refinement) principle, the endogenous high production of placental E2 during the end of pregnancy, and the fact that lasting effects of E2 exposure are much more likely to occur during the periconceptual period, this potential disadvantage was accepted. We thereby hypothesized that both F_1_ sampled (1) at 10 days of pregnancy and (2) at one year post parturition experienced the same E2 and/or perturbed uterine milieu due to E2 during the periconceptional period. Our findings clearly, in line with literature, highlight the periconceptual period as sensitive time window for the action of EDC^52^, and show that the level of effect of estrogenic EDC is at much lower concentration than currently presumed^31^. This may be of relevance in case of, e.g. oral food contamination, an unexpected conception during ongoing contraceptive treatment, or for women undergoing ovarian stimulation protocols during stimulated cycles in routine ART programs.

Most surprisingly, *CDKN2D* was hypomethylated at CpG − 200 and CpG − 190 in both liver of F_0_ sows and blastocysts of F_1_, whereas these sites were found to be hypermethylated in the liver of F_1_ offspring at one year of age. Likewise, while CpG +128 *PSAT1* was hypomethylated in both liver of F_0_ sows and blastocysts of F_1_, this CpG was similarly hypermethylated in the liver of F_1_ offspring at one year of age. In mice, the exposure to estrogenic EDC such as diethylstilboestrol (DES) and bisphenol-A (BPA) during pregnancy or neonatally has been shown to alter epigenetics marks in the uterus and liver, and has been linked to breast cancer via altered expression of histone methyltransferases^53–55^. One of the few studies investigating methylation changes upon exposure to estrogenic EDC in both F_0_ and F_1_ evidences DNA methylation changes of human foetal lung and placenta tissue upon *in utero* nicotine smoke exposure^56^. This epigenetic fingerprint is in line with the methylation patterns of *CDKN2D* and *PSAT1* observed in the F_0_ tissues and the F_1_ blastocysts. The switch within the F_1_ during development, namely from hypomethylation in blastocysts to hypermethylation at one year of age remains puzzling. From a regulatory point of view, epigenetic effects are frequently observed as opposing phenotypes, e.g. over- and undergrowth, and resemble a disequilibrium or dysregulation. Thus, if DNA methylation leads to gene expression changes of *CDKN2D* and *PSAT1* over time from foetus to adult, the observed hypermethylation in adult gilts could resemble the consequence of a dysregulated response. Unravelling the physiological DNA methylation changes during foetal and post-partum development over time, including the phase of puberty, may substantiate this assumption.

It seems highly unlikely that *CDKN2D* and *PSAT1* are the only two genes in F_1_ targeted by the periconeptional E2 treatment. A genome-wide, high resolution DNA methylome screening might unravel further differential DNA methylation to functionally link the molecular state of the blastocysts with the sex-specific metabolic phenotype observed in the adult F_1_^5,25^. Our data could therefore be indicative of the priming for development of disease later in life^50^.

In summary, the gestational effect of E2 was measurable as a tissue-specific molecular fingerprint on gene expression and DNA methylation including intergenerational epigenetic changes. The rising prevalence of endocrine related disorders in the recent past cannot be solely attributed to genetic factors and, therefore, our findings are relevant for human health^50,51,53–55^. Future studies should emphasize on studying short-term, intergenerational, as well as transgenerational tissue-specific effects to gain further understanding of the mechanism by which exposure to estrogenic EDC affects DNA methylation. Further, it should be investigated whether the absence of a clear phenotypic change upon the exposure to low-dose estrogenic EDC implies that the observed molecular effects are only transitory and thus non-alarming, or mirror latent fingerprints that may lead to a functional effect later in time. The latter implies that epigenetics could serve as novel sensitive parameter for risk assessment of estrogenic EDC.

## Materials and Methods

### Animals

The animal trial was performed with German Landrace sows as previously described^25^. Experimental procedures are described in detail in the Supplementary Text S1. In brief, animals were randomly distributed in four groups receiving different doses of E2 (0.05, 10 and 1000 µg/kg body weight/day, and a control ethanol carrier). The doses of E2 were orally applied to sows (n = 4–6 per treatment group) twice a day, to guarantee lasting elevated circulating estrogen concentrations, during the whole period of pregnancy. On day 7 of pregnancy, the average weight ± standard deviation of the sows was 218 ± 41, 239 ± 44, 239 ± 40, and 221 ± 40 kg for control, ADI, NOEL, and HIGH, respectively. On day 107 of pregnancy, the average weight ± standard deviation was 273 ± 29, 293 ± 34, 295 ± 31, and 286 ± 30 kg for for control, ADI, NOEL, and HIGH, respectively. The average age ± standard deviation of the sows at delivery was 29 ± 9, 37 ± 10, 31 ± 6, and 27 ± 3 months for control, ADI, NOEL, and HIGH, respectively. The average number of living offspring ± standard deviation was 8 ± 4, 10 ± 2, 10 ± 4, and 10 ± 3 piglets for for control, ADI, NOEL, and HIGH, respectively. The average birth weight of the offspring ± standard deviation was 1.8 ± 0.7, 2.0 ± 0.5, 1.7 ± 0.6, and 1.5 ± 0.4 kg for for control, ADI, NOEL, and HIGH, respectively. Piglets were weaned at 21 days of age and followed up to 1 year of age. Sows were subjected to an additional similar treatment during a following conception and slaughtered one hour after the last treatment on day 10 of this pregnancy, whereby each sow received the same treatment dose in both pregnancies. Embryos were recovered by uterine flushing upon slaughter. For the subsequent analysis, embryos with a minimum DNA content of 125 ng and an average DNA content of 18.1 ± 1.5 ng/µl (mean ± SEM) for the local DNA methylation analysis were selected randomly, and distributed as follows: control, ADI, NOEL, and HIGH dose, 9 embryos (4 females, 5 males), 9 embryos (5 females, 4 males), 10 embryos (4 females, 6 males), and 8 embryos (4 females, 4 males), respectively. The female offspring were slaughtered at one year of age (n = 6 per treatment group) during the luteal phase (10–13 days following estrus behaviour) after at least three ostroes cycles after onset of pubery. All experiments and samplings were conducted in accordance with accepted standards of humane animal care and were approved by the District Government of Upper Bavaria, reference # 55.2-1-54-2531-68-09.

### DNA/RNA extraction, quantification and quality assessment

Tissue samples and embryos for nucleic acid extraction were collected after slaughter, snap frozen in liquid nitrogen and stored at −80 °C until further analysis. Total DNA and RNA of tissues was extracted with the AllPrep DNA/RNA Mini Kit (Qiagen, Hilden, Germany), following the manufacturer’s instructions. Due to high fiber content of heart and skeletal muscle, total DNA and RNA was extracted using a specialized AllPrep Universal DNA/RNA/microRNA kit (Qiagen, Hilden, Germany), following the manufacturer’s instructions. After extraction, RNA and DNA was quantified and the quality was assessed as explained previously^51^. Total DNA and RNA of embryos was extracted with the AllPrep DNA/RNA Micro Kit (Qiagen, Hilden, Germany), according to manufacturer’s instructions with slight modifications (SI Text). Purity and quantity was assessed spectrophotometrically using the NanoDrop 1000 (peqLab, Erlangen, Germany). Additionally, RNA quantity of embryos was determined using the Qubit (Invitrogen) with the Qubit™ RNA BR Assay.

### Global DNA methylation analysis

Global DNA methylation was assessed by combining enzyme digestion with pyrosequencing using the LUminometric Methylation Assay (LUMA) as described previously^57^, with minor modifications due to the PyroMark Q48 Autoprep System (Qiagen): the complete reaction digest was transferred to each well of a PyroMark Q48 Autoprep Disc, and 6.5 µl of PyroMark Annealing Buffer was added automatically by the instrument. The nucleotides were added to the cartridge chambers as follows: 50 μl dATPαS and 50 μl water to “A”, 50 μl dTTP and 50 μl water to “T”, 50 μl dCTP and 50 μl dGTP to “C”, and 100 μl water to “G”. Pyrosequencing was performed with the nucleotide dispensation order 5′-ACTCGA-3′.

### Gene expression analysis

The cDNA was synthesized as described previously^51^. Target gene specific primers were designed using the publicly available NCBI Primer Blast software and purchased from Microsynth (Balgach, Switzerland). Target genes were selected based on their previously reported E2-mediated effects. The PubMed database was used to search papers with the following keywords: ‘estradiol-17β’, ‘transcriptomics’, ‘gene expression’, ‘pregnancy’, ‘cancer’, and ‘tumor suppressors’. All primers were tested for specificity by melt-curve analysis and gel electrophoresis. Primer sequences for all target genes can be found in (Supplementary Table S1). To determine gene expression, a high throughput gene expression platform was used based on Dynamic Array™ microfluidic chips (Fluidigm 96.96 Dynamic Array 15 IFC, BioMark™ Systems). The mRNA expression of selected target genes and four reference genes was measured at an annealing temperature of 60 °C and according to the Fluidigm Advanced Development Protocol 14 (SI Text). Cq values were obtained using a single threshold, a linearity range of 7 to 21 Cq was selected for further analysis, and relative quantification of the mRNA levels was performed with the 2^−ΔΔCq^ method^58^. The geometrical mean of four reference genes (*HPRT1*, *TBP*, *RPL4* and *ActB*) was used for normalization (Supplementary Table S2).

### Design of DNA methylation assays

The region of interest for the DNA methylation assay of *CDKN2D* was selected based on previous evidence of a putative promoter region in *Homo sapiens* lying within the region −774 bp to +18 bp from the translational start site^26^. This region was aligned with the *CDKN2D* sequence of *Sus scrofa* with the NCBI Blastn tool revealing 77% identity. The pyrosequencing was performed on CpN sites −269 to −182 for methylation analysis (Supplementary Figs S2 and 3). The region of interest for the DNA methylation assay of *RASSF1* was also selected based on previously documented evidence^16^. Similar to *Homo sapiens*, the *RASSF1* gene in *Sus scrofa* revealed a large CpG island spanning −304 bp to +349 bp from the translational start site (Supplementary Fig. S3). The CpG islands indicated in the figure were identified using a publicly available software (Meth Primer)^59^, with the following criteria (Island size > 100, GC % > 50.0, Obs/Exp > 0.6). Pyrosequencing was performed on CpG sites −137 to −55 (Fig. 3) in the 5′UTR. For *PSAT1*, thirteen CpN sites from +119 to +175 in the coding region of the first exon were selected for methylation analysis (Fig. 3).

### Bisulfite pyrosequencing

The bisulfite pyrosequencing was performed as explained earlier^51^, with slight modifications (SI Text). The primers for the PyroMark assays were designed using the PyroMark Assay Design Software 2.0 (Qiagen, Hilden, Germany) (Supplementary Table S3). The annealing temperatures were 56 °C, 58 °C, and 56 °C for the *CDKN2D, PSAT1*, and *RASSF1* assays, respectively. The product specificity was validated by gel electrophoresis and CpN methylation was quantified using the PyroMark Q48 Autoprep System (Qiagen) and the PyroMark Q48 Advanced CpG Reagents (Qiagen). Methylation values [%] were calculated with the PyroMark Q48 Autoprep 2.4.1 Software (Qiagen).

### Statistical analysis

All statistical analyses were performed using the IBM SPSS Statistics Software version 22 (IBM, 295 Böblingen, Germany). In order to determine the effects of E2 on gene expression, global DNA methylation and gene specific DNA methylation, a one-way ANOVA was used to detect significant differences followed by Dunnett’s post hoc test. To detect site-specific changes, and as reported previously^60,61^, each individual CpN site has been analysed by a one-way ANOVA followed by Dunnett’s post hoc test. The correlation analyses were performed using Pearson correlation. Statistically significance is displayed in the figures by *, **, and *** indicating statistical significance with respect to the control group at p < 0.05, p < 0.01, and p < 0.001, respectively.

## Electronic supplementary material

Supplementary Dataset

Supplementary Data
