# Supplementary material for: Gestational oral low-dose estradiol-17β induces altered DNA methylation of CDKN2D and PSAT1 in embryos and adult offspring
Source: Sci Rep. 2018 May 10;8:7494. doi: 10.1038/s41598-018-25831-9 (PMC5945594; doi:10.1038/s41598-018-25831-9)
Supplement: Supplementary file 1 — Supplementary Dataset [file 41598_2018_25831_MOESM1_ESM.doc]

**Supplementary Dataset**

Gestational oral low-dose estradiol-17β induces altered DNA methylation of CDKN2D and PSAT1 in embryos and adult offspring.

Vera A. van der Weijden^1 #^, Veronika L. Flöter^1, 2 #^, Susanne E. Ulbrich^1, 2*^

^1^ETH Zurich, Animal Physiology, Institute of Agricultural Sciences, Zurich, Switzerland

^2^Physiology Weihenstephan, Technical University of Munich, Germany

^#^Equally contributing authors

*Corresponding author:

Susanne E. Ulbrich

Animal Physiology, Institute of Agricultural Sciences, ETH Zurich

Universitätstrasse 2, CH-8092 Zurich, Switzerland

Phone.: +41 787198510

Mail: seu@ethz.ch

Major Category: Biological Sciences

Minor Category: Developmental Biology

**Detailed overview of the animal experiment**


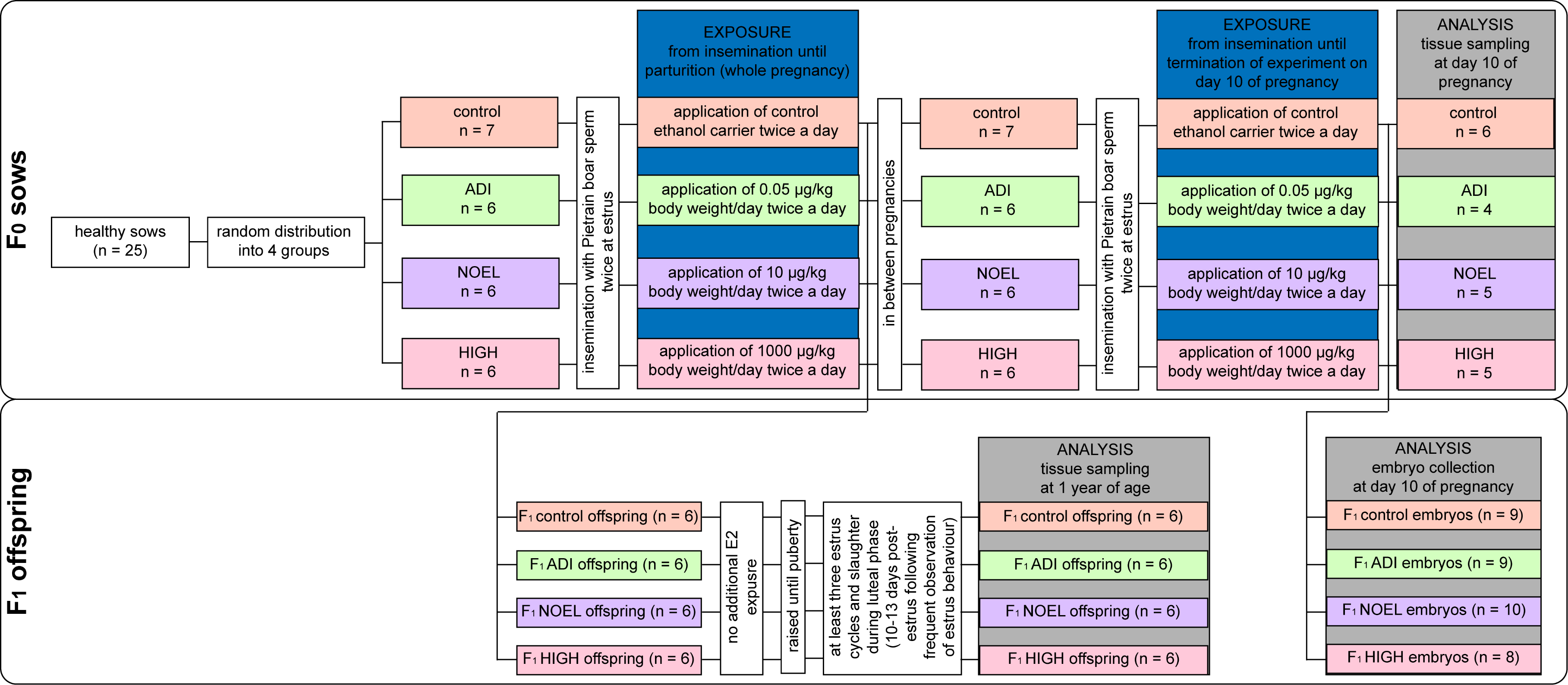
**Fig. S1. Graphical overview of the animal trail.** Control, ADI, NOEL, and HIGH represent the four different doses of 0, 0.05, 10 and 1000 μg/kg body weight/day, respectively. The exposure periods are marked by the blue boxes and sampling of tissue and embryos is marked in the gray boxes. The number of F_0_ sows, 1 year old F_1_ or day 10 F_1_ embryos indicate the number of analysed individuals.

**Supplementary Text S1**

**Detailed additional explanation of the animal experiment**

The confounding factor have been tried to be overcome as best as possible by randomization and standardization. However, due to management reasons, some challenging procedure, which are detailed below, could not be solved differently.

**Breeding**

Both sows and boars (F_0_) were each from the same race (landrace and pietrain, respectively), but were neither genetically identical nor arising from a specific strain, nor related by heredity. Therefore, the animals in this trail had a very heterogeneous breeding background. They sows were randomly allocated to the four treatment groups. They were between 21-42 months of age, had a body weight of 150 – 280 kg at insemination and had an uncomplicated own breeding history with different numbers of litters. All sows had previously been bred at least once. Due to the worldwide lack of protocols for freezing boar sperm up to date, sperm for artificial insemination in pigs was only available directly after sperm collection at a local breeding station. Given the nessesity to use fresh sperm and the limited production by only one boar, the sows were randomly allocated to one of six breeding boars. The latter were of the same breed and randomly selected. Only healthy sows and sperm from healthy boars were included in the study. The treatment ended at the day of parturition. The offspring were born and weaned at 21 days of age and were randomly selected to either be slaughtered at day 63 [1] (not part of this study) or kept until one year of age (F_1_). After the onset of puberty of these F_1_, at least three estrus cycles were observed by determining faecal progesterone [2]. Then, randomly selected female offspring per sow (F_0_) were slaughtered during the luteal phase of the cycle (approximately cycle days 10-13).

The sows (F_0_) then underwent treatment again after repeated breeding, which were irrespective of the experimental trial. This was done solely due to management reasons and led to a random number of parturitions randomly distributed across the treatment groups, at least twice per sow. The sows (F_0_) entered the study during November – December 2009. For the final breeding regarding the second E2 treatment, the same sows (F_0_) were allocated to the same treatment group as in the first E2 treatment. Each sow was slaughtered 10 days after insemination. This time, the sows were bred to the same boar. Therefore, all embryos (F_1_) collected at day 10 are progenies of the same boar.

**Housing and feeding**

All animals were housed indoors and were held under appropriate standardized sanitary conditions. The water was allocated through a circulating system. All feed taken from a same batch was randomly allocated to all animals. Next to Zearalenon (ZEN) (1.6 µg/kg feed), only Deoxynivalenol (DON) as possible mycotoxin feed contaminant was determined in the feed (97.7 µg/kg) and was considered to be below the critical exposure level to cause a confounding effect. Thereby, all animals were expected to have contact to either none or the same minimal exogenous EDC contamination.

The sows were trained to eat bread rolls for several consecutive days. The bread rolls were carefully supplied with E2 dissolved in ethanol carrier in the lab and allocated to individual plastic bags per treatment group per feeding twice daily. All bread rolls were stored in a freezer in different storage drawers per treatment dose and thawn only shortly before feeding. It was assured that the ethanol did not drip from the prepared bread rolls to neither lose any ethanol carrier nor to contaminate any facility surface. The staff was advised to strictly avoid any bread roll cross contamination possibilities during the feeding procedure. The bread rolls were handled with different gloves per treatment group for each feeding. Throughout the entire experimental procedure, the animals were housed either in single boxes or in groups related to their respective treatment. The bread rolls were fed manually and individually to the sows and it was visually observed that the sows had eaten the bread rolls at all times during the experiment. Before leaving the group and stable compartment, respectively, the gloves were discarded with the plastic bags in order not to contaminate any stable housing equipment.

The purpose of the study was to mimic a continuous exposure during pregnancy via the route of oral uptake. Due to the elimination kinetics, we knew that E2 levels were within the maximum peak hight one hour after the oral E2 uptake and then rapidly cleared from the blood due to liver metabolism [3]. Therefore, we allocated the daily dose into two parts in order to have as long circulating E2 (and metabolites) as possible within the practical handling of feeding twice per day. Elevated plasma concentrations of free circulating E2 (high dose) and E2 metabolites (high dose and NOEL dose) were observed over the 12 hours intervall between feedings, thereby mimicking the continuous exposure as intended. The bread roll was fed before the normal feeding in order to standardize the amount of feed in the intestinal tract of the sow as best as possible. The direct E2 effect was assessed at 1 hour after feeding, assuming that the circulating levels of E2 would be within the maximum range (as known from the former elimination kinetics), while gene expression changes due to the treatment offered the opportunity to be assessed.

There was not any treatment during the period of lactation as the treatment feeding terminated at parturition.

**Treatment effects and methylation in day 10 embryos**

Neither litter size, nor litter sex ratio, nor embryo sex ratio was affected by the treatment [3] (Flöter et al., under review). The F_1_ female offspring were randomly selected from the litters of at least 4 different sows per treatment group. The same randomisation procedure was applied for selecting the embryos flushed from the uterus of the respective sows. The size of the embryos was 2.2 mm ± 0.1 mm on average (n = 230) and there were no differences between treatment groups (p = 0.80).

We aimed at finding epigenetic changes in the offspring through E2 exposure during pregnancy. We hypothesized that there are already alterations early during pregnancy that might contribute to the origin for the observed lasting effects. Regarding such epigenetic marks like DNA methylation, huge remodeling occurs before day 10 [4, 5]. As known from assisted reproductive technology (ART) [6] these indicate a potential sensitive time for exogenous stimuli to modify these marks. In both mice and bovine, there is active and passive demethylation. In mice, the *de novo* methylation starts after the morula stage, whereas in bovine this already starts after the 8-cell stage [7]. In addition, in day 8 porcine blastocysts, the embryos show a high degree of methylation in the inner cell mass and relatively stable degree of methylation in the trophectoderm compared to early blastocysts (day 5) [8]. On day 10 of porcine embryo development, the blastocyst is already shortly prior to elongation, i.e. an expanded blastocyst. DNA methylation marks are erased before the blastocyst stage, and that *de novo* methylation has already started to take place. The continues gestational exposure during both demethylation and *de novo* methylation was of specific interest. For our analyses, we wanted to select a time where the marks have reached a relatively stable level in both the embryoblast and the trophectoderm, which is the case at day 10 [4, 9]. In addition, at day 10 low endogenous estrogen concentrations prevail [10]. It is the time point before the embryo produces estrogens. Using day 10, the effect of the applied exogenous E2 doses on the sows and embryos without any embryonic-derived contribution would be obeserved. Therefore, this time phase was selected to resemble best the situation in other species including women.

**Supplementary Methods**

**DNA/RNA extraction, quantification and quality assessment**

All tissues were homogenized using the MagNA Lyser instrument and 1.4-mm (diameter) ceramic beads (Roche Life Science, Mannheim, Germany) were used. For RNA and DNA extraction of embryos, 700 µl of Buffer RLT Plus supplemented with 1% β-mercaptoethanol was added to the frozen embryo. Disruption was achieved by pipetting up and down and by a single brief vortexing. Homogenization was performed using a syringe and needle. After centrifugation of the lysate using a DNA spin column, the column was stored at 4°C, while the flow-through was processed following the protocol for “purification of total RNA containing small RNAs from cells”. In order to improve RNA purity, the column was incubated with Buffer RPE at step D3 and D4 before centrifugation for 4 min and 2 min, respectively. RNA elution was repeated using the first eluate to increase the final concentration. The DNA was purified subsequently. Samples were immediately put on ice. RNA and DNA samples were stored at -80 °C and -20 °C, respectively.

**Gene expression analysis**

The mRNA expression of selected target genes and four reference genes was measured at an annealing temperature of 60°C and according to the Fluidigm Advances Development Protocols 14. In brief, before introducing the samples to the Fluidigm platform, the cDNA was pre-amplified using the TaqMan PreAmp Master Mix (Applied Biosystems) with the following conditions: 95°C for 2 min and 14 cycles of 95°C for 15 sec and 60°C for 4 min. For each sample, 1.25 μl assay mix consisting of a forward and reverse primer (concentration 500 nM), 2.5 μl 2xABI preamp mix and 1.25 μl cDNA was subjected to pre-amplification. Pre-amplified cDNA was cleaned up with Exonuclease I and 1:5 diluted. The sample mix consisted of 4 μl 2 × TaqMan Gene Expression Master Mix (Applied Biosystems), 0.4 μl 20 × DNA Binding Dye Sample Loading Reagent (Fluidigm), 0.4 μl 20× EvaGreen DNA binding dye (Biotium), 1.7 µl 1× DNA Suspension Buffer, and 1.5 μl pre-amplified cDNA. The assay mix consisted of 4 μl Assay Loading Agent (Fluidigm), 3.6 μl of 20 μM Forward and Reverse Primer Mix and 0.4 µl 1× DNA Suspension Buffer. For the qPCR run, 5 μl of sample and assay mix was loaded on the Fluidigm chip. To ensure specific product amplification from the qPCR, a melting curve analysis was performed.

**Bisulfite pyrosequencing**

In brief, for all tissue samples 1 µg of DNA was subjected to bisulfite conversion with the EpiTect Bisulfite Kit (Qiagen, Hilden, Germany) according to manufacturer’s instructions, whereas 125 ng of input DNA was used for each embryo. The concentration of bisulfite converted DNA (bcDNA) was determined with the QuantiFluor® ssDNA System (Promega), according to manufacturer’s instructions, and used as a template for PCR amplification using the PyroMark PCR kit (Qiagen, Hilden, Germany) and a Labcycler (Sensoquest, Göttingen, Germany).

To assess the accuracy of the measurements, a set of control DNA with known methylation levels (0 %, 50 % and 100 %) was established using whole genome amplification and *in vitro* methylation of genomic DNA according to the guidelines described previously [11].

Table S1. Target Genes and Primer Sequences

| **Gene Name** | **Gene ID** | **Primer Sequence**  **5’-3’** | **Amplicon Length (bp)** |
| --- | --- | --- | --- |
| *ALAS2* | 100518817 | ATCCTGCCAGGGTGTGAGAT  AATTTTGGGTGTCACGGGGT | 159 |
| *BPGM* | 106506774 | AAAGCTCCTGGCGTCTCAAT  GGGCGGTGGGGTTATATTGT | 131 |
| *BUB1* | 100627208 | TGCGGAAACCCATTTACCGA  AGCTCTGAACCCTGCTTCTT | 138 |
| *KIF4A* | 100152604 | TGTGGGTGCAGGAGACAAAA  GCCCAAGCTCTCCTTGTCTT | 96 |
| *MPO* | 100517120 | GCATCATCTGCGACAACACG  TGCAGTTGACAAAGTCCCGA | 90 |
| *MMP8* | 100523811 | TCCATGGACCCAGGTTATCCC  AAGGAAGAAGTGATGTTGCTGGAA | 96 |
| *CDC42EP3* | 100521443 | GCCAGTGCTCAAAAATGCCA  CACGACTGGCTCACAGCTAA | 148 |
| *CCDC34* | 100518823 | GCAGGAGGGAGCTGGTTATG  CAAACTGGCAGCTCTGGGAT | 158 |
| *CDC42EP4* | 100517117 | GCATTTCTGATGTGACCGCC  AGAAGGGGTGGCGTTTAGTG | 145 |
| *CCNI* | 100523679 | GCTTCAGAAAGCACAGATGGAG  CGGAGTTTAGAGGCAGGGC | 101 |
| *CDC42BPA* | 100511216 | AAGAAGGAATCGGTCGCACA  AGGGCATATCTGTTGCTCGG | 169 |
| *GADD45B* | 100621090 | TCCTTGGAACTGTCGTGTGG  CTCACCGTCTGCATCTTTTGC | 109 |
| *GADD45A* | 733669 | CATGATCCAGGAGCCCGAAG  TCCATCCTGCGTGGTTCTTT | 128 |
| *GAS1* | 100516459 | TCTATGTGCAAAGGGGCTGG  GCGCGGATACCGAGAGTAAA | 133 |
| *HSD17B7* | 100155418 | ATTCCAGTGTGGTGTGTCCAG  AAGCGCAACAGCCATATCATT | 107 |
| *Lhcgr* | 407247 | TCTGCCATCTTTGCTGAGAGT  TCTGGTTCGGGAGCACATTG | 92 |
| *Actg2* | 100520667 | TTGCCATTCAAGCTGTGCTC  GGCAGTGCGTATCCCTCATAG | 115 |
| *BTG2* | 100048932 | CTGCCGCTGTAGGTTTCCTC  AGTGGTGTTTGTAGTGCTCTGTC | 120 |
| *RERG* | 100155884 | TCCCACCCTCGAATCAACCT  CATCGCATGTGTCCTTCCCT | 120 |
| *P53* | 100152579 | GCTTTGAGGTGCGTGTTTGT  CACGGATCTGGAGGGTGAAAT | 195 |
| *PTEN* | 100156264 | TGCAGAGTTGCACAGTATCCTT  ACACCAGTTCGTCCCTTTCC | 152 |
| *RB1* | 100151828 | CTTGGCGCTCAGATTCACCT  TTGGGGGATCTTAAAGGAGAAAGA | 160 |
| *LATS1* | 100152165 | ATTAGAGCGGAGAGCTGCAC  AACTGTGTATAGCCTGTTCGT | 111 |
| *HIC1* | 100525779 | TCTTCCTGTGGTTCGTTGGC  CCAGCAGGGAGATTCACAGT | 136 |
| *CDKN2C* | 100515210 | ACTGCGCTGCAGGTTATGA  CTGGCTGCATCGTGAATGAC | 119 |
| *CDKN2D* | 100525560 | CGCCAGTATCGATATGCTGCTA  CTGCCAAACATCATGACCTGC | 171 |
| *CDKN1A* | 100152215 | GCAGACCAGCATGACAGATTT  TGTCCCCTTGTTTCCAGCAG | 109 |
| *CDKN1B* | 397584 | GCACTGGGATAAGGAAGCGA  AGCAGTTTACGTTTGACGCC | 161 |
| *NF2* | 100520699 | TCCTTCCTCCAAAACACCCTG  ATGTTCTGGAACCCTTGGTGG | 157 |
| *SFRP1* | 100621622 | TGTGTGTCCTCCATGTGACAA  TTGATCTTCATCCTCAGGGCAAA | 90 |
| *SFRP5* | 100153176 | CAACGACCTCTGCATCGCT  AGGCGCATTTTGACCACGAAG | 150 |
| *DAPK1* | 100516103 | CCACAGCACAGGCGAGATAAA  ATAGCAACTTCCCCAGCACG | 104 |
| *MGMT* | 100155050 | CAGCAAGAGTCGTTCACCAGA  CAGGATTGCTCCTCATGGCT | 148 |
| *MeCP2* | 100524967 | GGAATGTTAGGGCTCAGGGAA  CCCTCTCGGTCGTCTTTCTT | 115 |
| *MBD1* | 100156150 | GAACCTAGAGCGGCGGAAG  TCCTGTGGGGCTCTGGTAAT | 186 |
| *RPL4* | 100156150 | TTCAAGGCTCCCATTCGACC  GCACTGGTTTGATGACCTGC | 110 |
| *TBP* | 110259740 | CTGTGTGGTGCAAGGATGGA  GCCCGAATAGCAGCACAGTA | 146 |
| *HPRT1* | 397351 | TGCCGAGGATTTGGAAAAGGT  CACACAGAGGGCTACGATGT | 118 |
| *PSAT1* | 100154160 | ACCTGATTGGCCTGAAAGCA  CAGGTGCTCGGATCTGGAAT | 154 |
| *GSTA4* | 100152951 | GTGTTCGAAAAGGTTTTACGGGG  GAGGTGAGGAAAGGCAGACAG | 141 |
| *IL-6* | 100628202 | TCTGGGTTCAATCAGGAGACC  CGGCCTCGACATTTCCCTTAT | 118 |
| *RASSF1* | 100516830 | AGTGGGAGACACCTGACCTT  GGAGCCGTCTTTGTTAAGGC | 107 |
| *Diablo* | 100155744 | CTGCATACCAAACTGGAGCG  CTGGGAAAGCTGGCGTACTT | 98 |
| *DNMT1* | 606746 | ATAAGATTGAGACCACTGTTCC  CTACTTGTTCCACCACAAACTG | 99 |
| *DNMT3a* | 100037301 | CAAAGTGAGGACCATTACTACGA  CCGAACACCCTTTCCATCTC | 123 |
| *DNMT3b* | 100302109 | TAAGTTACACTCAGGATTTGACGG  AGGTACTGTTGTTATTTCGGGT | 150 |
| *ESR1* | 397435 | CCATCATTTTGCTTAATTCTGGAG  ATGCCT TTGTTACTCATGTGCCTG | 194 |
| *ESR2* | 396697 | TCTGGAAATCTTTGACATGCTC  AGGATACATACTGGAGTTGAGG | 118 |
| *CCDC80* | 100516578 | ATCACCATCTTTGGTCCTGTC  TGGGTTTCTCGTTGTCTAACTG | 73 |
| *BGN* | 397396 | GAACTGCATCGAGATGGGTG  AGTTCGTTCAGAGTCTCGGG | 147 |
| *ADH1C* | 100512615 | GGATTGACACTATGATTTCTGCC  GGTACTCCTACAATGACGCT | 73 |
| *HOXA10* | 397290 | AAAGAGCGGCCGGAAGAA  ACGCTGCGGCTGATCTCTAG | 120 |
| *SPARC* | 100514136 | CGAGTTTGAGAAGGTATGCAG  CAAGGTCCGATGTAGTCCAG | 129 |
| *ActB* | 414396 | GATGACTCAGATCATGTTCGAGAC  CAGAGTCCATGACAATGCCA | 113 |
| *AR* | 397582 | AGAGTGCCCTATCCAAGTCC  GTAGGTTCCAAACGCATGTC | 98 |
| *ESRG* | 100622056 | GCATATTCCAGGCTTCTCCA  TCTGCATAGACAAGTTCATCCT | 132 |

Table S3. Primer Sequences for Bisulfite Pyrosequencing

| **Gene Name** | **Gene ID** | **Primer Sequence**  **5’-3’** | **Sequencing Primer**  **5’-3’** | **Amplicon**  **Length (bp)** |
| --- | --- | --- | --- | --- |
| *CDKN2D*  (CpN -270 to -183) | 100525560 | GGGTGAGTTGGGGGAGAT  CCAACTAACTCCTCCCCCTATCA | GAGTTGGGGGAGATT | 126 |
| *PSAT1*  (CpN +121 to +171) | 100154160 | GAGTAGGAGTTGGTTGTAAGAT  ACCCCCAAATTAACCACCTACATCAA | AGTTGGTTGTAAGATTTG | 120 |
| *RASSF1*  (CpG -137 to -55) | 100516830 | AGGGATGAAGGGAGGTAG  ACCCAAACTCCTACTAACTCTAAAC | TTTGGGTGTTGGGTA | 244 |


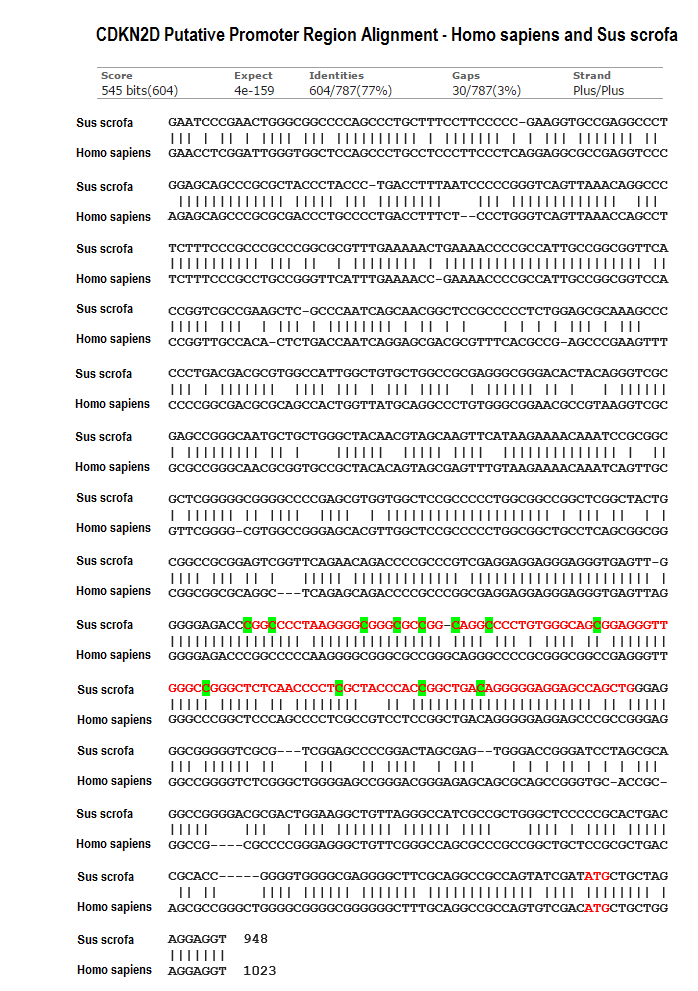


**Fig. S2.** Homo sapiens *CDKN2D* putative promoter region aligned with Sus scrofa revealing 77% identity. Bisulfite pyrosequencing was carried out on the sequence shown in red and CpN sites analysed are highlighted in green.


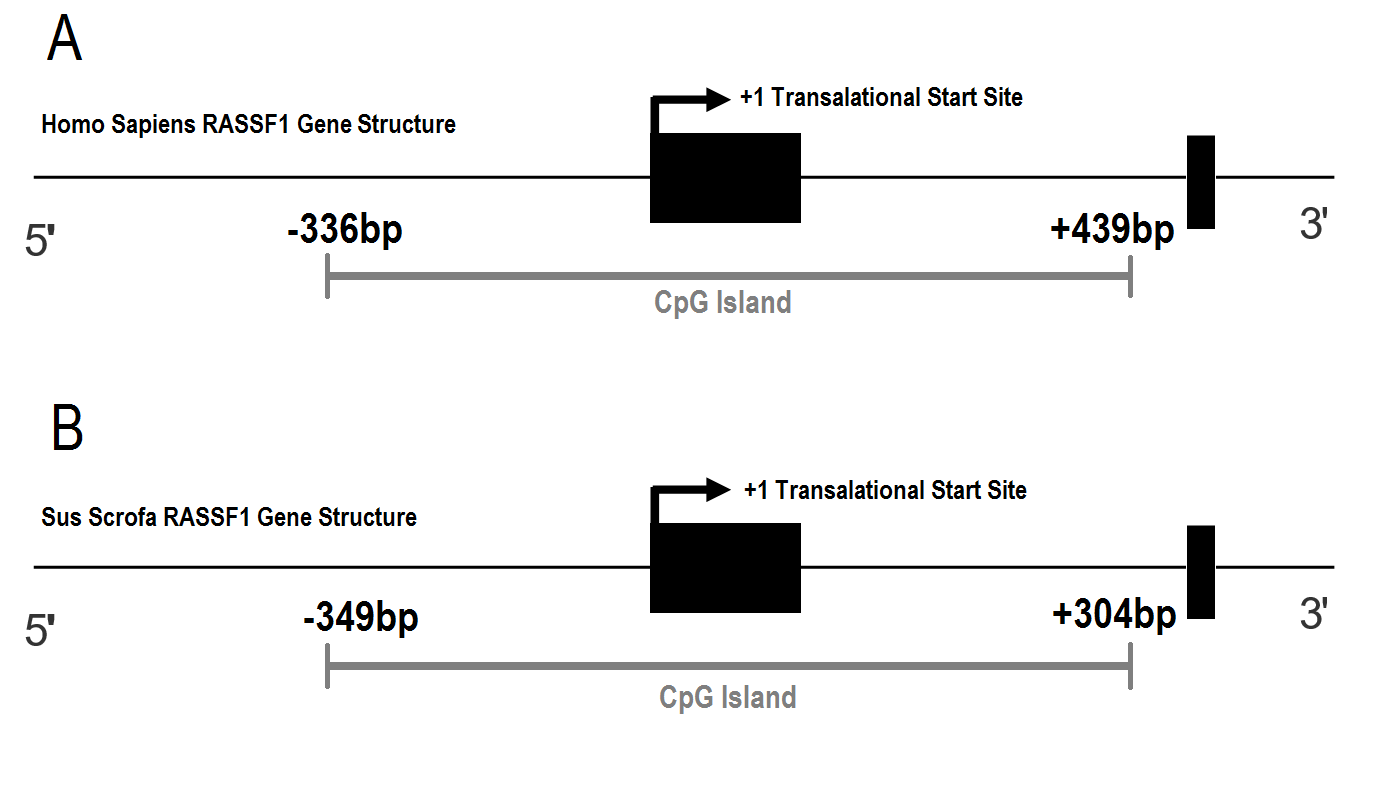


**Fig. S3.** ***RASSF1* Gene Structure and GC rich region** (A) Homo sapiens *RASSF1* gene structure and GC rich region identified previously by Starland-Davenport et al. 2010. (B) GC rich region identified in Sus scrofa *RASSF1* gene structure.

Starlard-Davenport, A., et al.*, Mechanisms of epigenetic silencing of the Rassf1a gene during estrogen-induced breast carcinogenesis in ACI rat*s. Carcinogenesis, 2010**.** 31(3): p. 376-381.


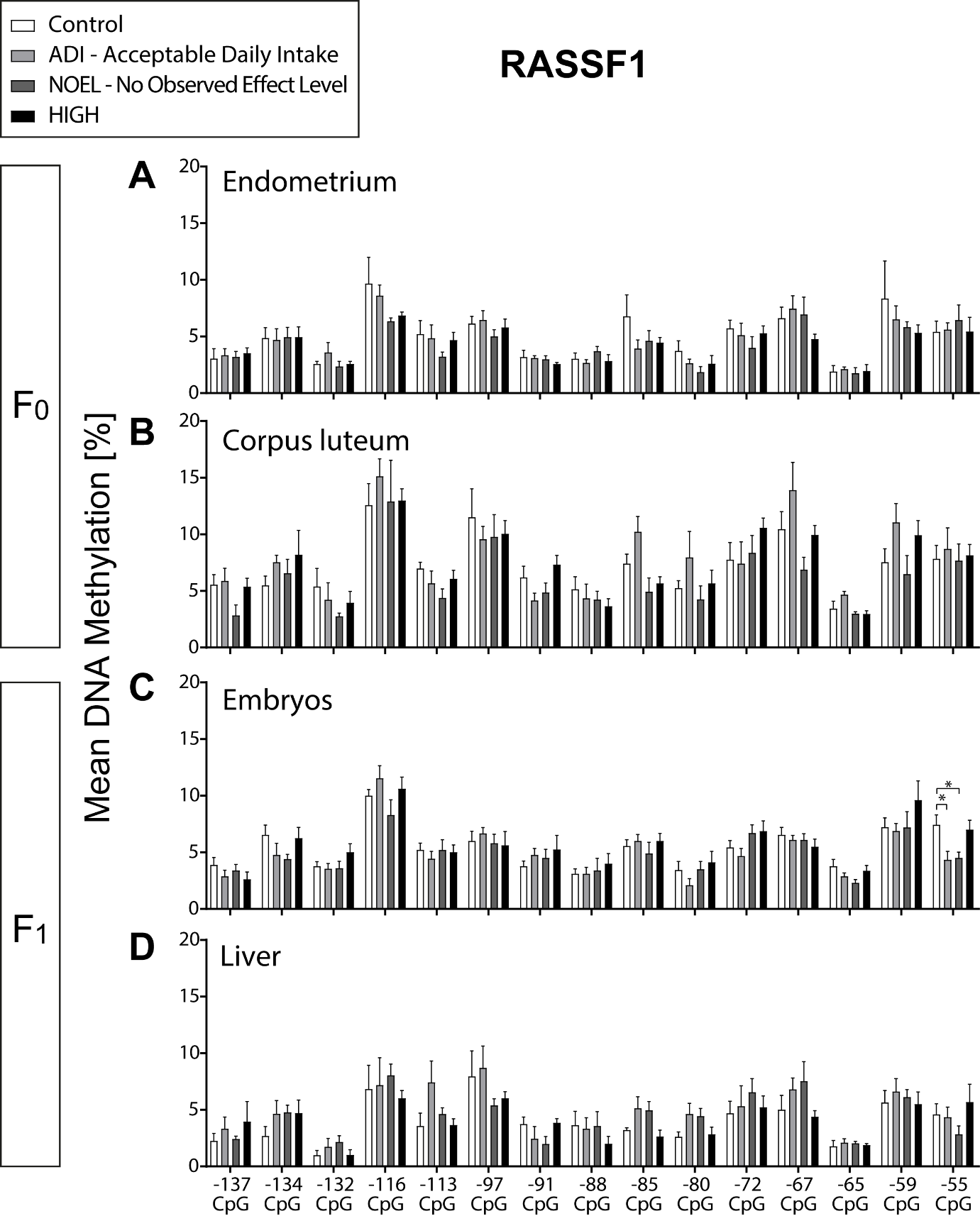


**Fig. S4.** **DNA methylation in *RASSF1* 5′ UTR.** (A) F_0_ Endometrium and (B) F_0_ Corpus Luteum, (C) F_1_ day 10 embryos and (D) F_1_ liver. Bar charts represent mean ± SEM of % DNA methylation per group in different shades of grey (0, 0.05, 10, and 1000 μg/kg body weight/day represented by ADI, NOEL and HIGH dose, respectively).

**References**

1. Pistek, V.L., et al., *HOXA10 mRNA expression and promoter DNA methylation in female pig offspring after in utero estradiol-17beta exposure.* J Steroid Biochem Mol Biol, 2013. **138**: p. 435-44.

2. Floter, V.L., et al., *Sex-specific effects of low-dose gestational estradiol-17beta exposure on bone development in porcine offspring.* Toxicology, 2016. **366-367**: p. 60-7.

3. Furst, R.W., et al., *Maternal low-dose estradiol-17beta exposure during pregnancy impairs postnatal progeny weight development and body composition.* Toxicol Appl Pharmacol, 2012. **263**(3): p. 338-44.

4. Fulka, H., et al., *DNA methylation pattern in human zygotes and developing embryos.* Reproduction, 2004. **128**(6): p. 703-8.

5. Oestrup, O., et al., *From zygote to implantation: morphological and molecular dynamics during embryo development in the pig.* Reprod Domest Anim, 2009. **44 Suppl 3**: p. 39-49.

6. Wrenzycki, C., et al., *Epigenetic reprogramming throughout preimplantation development and consequences for assisted reproductive technologies.* Birth Defects Res C Embryo Today, 2005. **75**(1): p. 1-9.

7. Dean, W., F. Santos, and W. Reik, *Epigenetic reprogramming in early mammalian development and following somatic nuclear transfer.* Semin Cell Dev Biol, 2003. **14**(1): p. 93-100.

8. Fulka, J., et al., *DNA methylation pattern in pig in vivo produced embryos.* Histochem Cell Biol, 2006. **126**(2): p. 213-7.

9. Reik, W., W. Dean, and J. Walter, *Epigenetic reprogramming in mammalian development.* Science, 2001. **293**(5532): p. 1089-93.

10. Magness, R.R. and S.P. Ford, *Estrone, estradiol-17 beta and progesterone concentrations in uterine lymph and systemic blood throughout the porcine estrous cycle.* J Anim Sci, 1983. **57**(2): p. 449-55.

11. Furst, R.W., et al., *Is DNA methylation an epigenetic contribution to transcriptional regulation of the bovine endometrium during the estrous cycle and early pregnancy?* Mol Cell Endocrinol, 2012. **348**(1): p. 67-77.
